# Has introduction of rapid drug susceptibility testing at diagnosis impacted treatment outcomes among previously treated tuberculosis patients in Gujarat, India?

**DOI:** 10.1371/journal.pone.0121996

**Published:** 2015-04-13

**Authors:** Paresh Dave, Bhavin Vadera, Ajay M V Kumar, Palanivel Chinnakali, Bhavesh Modi, Rajesh Solanki, Pranav Patel, Prakash Patel, Kirit Pujara, Pankaj Nimavat, Amar Shah, Sandeep Bharaswadkar, Kiran Rade, Malik Parmar, Sreenivas Achuthan Nair

**Affiliations:** 1 State Tuberculosis Cell, Commissionerate of Health, Medical Services and Medical Education, Ministry of Health & Family Welfare, Gandhinagar, Gujarat, India; 2 World Health Organization (WHO) Country Office for India, New Delhi, India; 3 International Union against Tuberculosis and Lung Diseases (The Union), South-East Asia Regional Office, New Delhi, India; 4 Department of Community Medicine, GMERS Medical College, Gandhinagar, Gujarat, India; 5 Department of Pulmonary Medicine, B. J. Medical College, Ahmedabad, Gujarat, India; 6 Department of Preventive & Social Medicine, Jawaharlal Institute of Postgraduate Medical Education & Research, Puducherry, India; 7 State Tuberculosis Training and Demonstration Centre, Near Tuberculosis and Chest Department, Civil Hospital Campus, Asarwa, Ahmedabad, India; Indian Institute of Science, INDIA

## Abstract

**Background:**

Revised National TB Control Programme (RNTCP) in India recommends that all previously-treated TB (PT) patients are offered drug susceptibility testing (DST) at diagnosis, using rapid diagnostics and screened out for rifampicin resistance before being treated with standardized, eight-month, retreatment regimen. This is intended to improve the early diagnosis of rifampicin resistance and its appropriate management and improve the treatment outcomes among the rest of the patients. In this state-wide study from Gujarat, India, we assess proportion of PT patients underwent rapid DST at diagnosis and the impact of this intervention on their treatment outcomes.

**Methods:**

This is a retrospective cohort study involving review of electronic patient-records maintained routinely under RNTCP. All PT patients registered for treatment in Gujarat during January-June 2013 were included. Information on DST and treatment outcomes were extracted from 'presumptive DR-TB patient register' and TB treatment register respectively. We performed a multivariate analysis to assess if getting tested is independently associated with unfavourable outcomes (death, loss-to-follow-up, failure, transfer out).

**Results:**

Of 5,829 PT patients, 5306(91%) were tested for drug susceptibility with rapid diagnostics. Overall, 71% (4,113) TB patients were successfully treated - 72% among tested versus 60% among non-tested. Patients who did not get tested at diagnosis had a 34% higher risk of unsuccessful outcomes as compared to those who got tested (aRR - 1.34; 95% CI 1.20-1.50) after adjusting for age, sex, HIV status and type of TB. Unfavourable outcomes (particularly failure and switched to category IV) were higher among INH-resistant patients (39%) as compared to INH-sensitive (29%).

**Conclusion:**

Offering DST at diagnosis improved the treatment outcomes among PT patients. However, even among tested, treatment outcomes remained suboptimal and were related to INH resistance and high loss-to-follow-up. These need to be addressed urgently for further progress.

## Introduction

Treatment outcomes of previously-treated smear-positive pulmonary tuberculosis (PT) patients have remained poor globally and in India. Among PT patients registered in the year 2011 in India, 71% were treated successfully as compared to 88% new smear-positive pulmonary tuberculosis (TB) patients [1]. One of the important reasons for the poor outcome is related to the high baseline rates of multi drug resistant tuberculosis (MDR-TB) in PT patients, estimated to be about 12–17% in previous studies [2]. The World Health Organization recommends that all PT patients be tested for presence of MDR-TB at diagnosis [3,4]. India has implemented the policy of testing all previously treated TB cases for MDR-TB upfront before initiation of treatment under Revised National TB Control Programme (RNTCP) since 2011 [5]. The implementation has gained momentum with increasing availability of rapid drug susceptibility testing (DST) through WHO-endorsed rapid diagnostics (WRDs) like Line Probe Assay (LPA) or Cartridge Based Nucleic Acid Amplification Test (CBNAAT) (Xpert MTB/RIF-Cepheid, Sunnyvale, CA, USA). This intervention is expected to improve early diagnosis of rifampicin resistance and its appropriate management. Also, the policy is expected to improve the treatment outcomes of PT patients as the patients with rifampicin resistance would be segregated and only those without rifampicin resistance are initiated on standardized, eight-month, retreatment regimen. A systematic evaluation of effect of offering rapid DST at diagnosis among PT patients has not been done yet in India. In this state-wide study conducted in Gujarat, India, we aimed to assess the proportion of PT patients who underwent rapid DST at diagnosis and the impact of this intervention on their treatment outcomes.

## Methods

### Ethics

The study protocol was reviewed and approved by the Institutional Ethics Committee of B. J. Medical College, Ahmedabad, Gujarat, and the Ethics Advisory Group of International Union against Tuberculosis and Lung Disease, Paris, France. The administrative permission for the study was taken from the State TB Cell, Government of Gujarat. Since the study involved a review of records with no direct interaction with the patients, ethics committees waived the need for informed consent from each patient. All the personal identifiers were removed from the database before analysis.

### Study design

Retrospective cohort study involving a review of records maintained routinely under RNTCP.

### Study Setting

The study was conducted in the State of Gujarat, situated in the western coast of India. The state has a population of 60.4 million with 57.4% of population residing in the rural areas. Gujarat RNTCP was one of the early implementers of the policy that all the PT patients are screened for rifampicin resistance using WRD before initiation of treatment and have been implementing this policy across all districts. In early 2013, there were two culture and DST laboratories with facilities for performing LPA and one CBNAAT laboratory in the state. Drug susceptibility testing was provided through LPA in 24 districts serving 50.3 million population and CBNAAT in 6 districts serving 10.1 million population.

Once PT patients are diagnosed at any health facility, the laboratory technician collects two sputum specimens from the patient and transports it to the nearest laboratory with availability of WRDs. The results of DST are communicated back to the referring health facility within a week. Those diagnosed to be having MDR-TB or rifampicin resistance are initiated on a 24-month standardized regimen for MDR-TB. Patients who do not have rifampicin resistance or an invalid test result or who were not tested are treated with a WHO-recommended, standardized, thrice-weekly intermittent regimen (previously known as category II treatment) for previously treated TB patients (*2H*_*3*_ *R*_*3*_ *Z*_*3*_ *E*_*3*_ *S*_*3*_ *+1H*_*3*_ *R*_*3*_ *Z*_*3*_ *E*_*3*_ *+5H*_*3*_ *R*_*3*_ *E*_*3*_ *; H-Isoniazid*, *R-Rifampicin*, *Z-Pyrazinamide*, *S-Streptomycin*, *E-Ethambutol; H*_*3*_ *R*_*3*_ *Z*_*3*_ *E*_*3*_ *S*_*3*_ *are given for 2 months followed by H*_*3*_ *R*_*3*_ *Z*_*3*_ *E*_*3*_ *for 1 month during intensive phase and H*_*3*_ *R*_*3*_ *E*_*3*_ *are administered for 5 months in continuation phase*). The treatment is delivered under the direct observation (DOT) of a treatment observer either from the health system or the community.

A standardized recording (using TB treatment cards and TB registers) and reporting system [6] exists under the programme to capture patient related data. Since 2012, in addition to the paper-based recording system, a case-based, web-based electronic notification system for TB patients named NIKSHAY (meaning ‘No TB’) has been centrally developed and being implemented in the state. Under NIKSHAY, the information in the treatment cards are captured in an online electronic database by trained data entry operator at peripheral health facility.

### Study population and Study period

All smear-positive previously treated TB (PT) patients registered for treatment under RNTCP in the State of Gujarat from 01 January 2013 to 30 June 2013 were included in the study. This included 30 district TB centres (reporting units) and 144 sub district level Tuberculosis Units (TU). The study was conducted between October 2013 and June 2014.

### Data collection

Data variables included TB number, age, sex, HIV status, smear results and site of disease and were sourced from NIKSHAY and validated using TB registers. Whenever there was a discrepancy between the two, the information from TB register was considered final. In addition, data on treatment outcomes were extracted from the TB registers. Data on DST status and its results were captured from ‘presumptive MDR-TB patient register’ maintained at each TB unit level. Data extracted from NIKSHAY were imported into EpiData Entry software (version 3.1, EpiData Association, Odense, Denmark) database and additional information on DST and treatment outcome were entered. Data were collected by Senior Treatment Supervisors, Senior TB laboratory supervisor of respective TB Units and DR-TB supervisor of respective district. Data entry was done by district data entry operator. The data were validated by comparing the aggregated reports under the programme on DST coverage, TB patients registered and treatment outcomes for each district. Feedback was shared with the districts on the number of records with discrepant data and the same was rectified by referring to the original records. The case definitions and treatment outcomes used in the study were as per national guidelines which are aligned with WHO recommendations. [7]

### Analysis

All district wise EpiData files were appended in EpiData analysis and a master database was created. The database was then analysed using EpiData (Version 2.2.2.182, EpiData Association, Odense, Denmark). Demographic and clinical characteristics of those who were tested for drug susceptibility were compared with those who were not tested using Chi square test. For the purpose of analysis, treatment outcomes were categorized to successful outcomes (cured and treatment completed) and unsuccessful outcomes (death, failure, loss to follow-up or default, transferred out and switched to Category IV (MDR-TB treatment) regimen. As a subset analysis, treatment outcomes were compared between INH resistant and INH sensitive patients.

Bivariate analysis was done to study possible association of age, HIV status, type of TB and DST status with treatment outcomes. Relative Risks (RR) and 95% confidence intervals (CI) were calculated. All these factors were included in a log-binomial regression model to assess the independent effect of rapid DST at diagnosis on treatment outcomes and adjusted relative risks were calculated. The multivariate analysis was done using STATA (version 12.1, TX, USA). P value ≤0.05 was considered statistically significant for all analyses.

## Results

A total of 6,454 previously treated smear positive pulmonary TB (PT) patients were registered for treatment. Of these, 541 patients did not have their details entered in NIKSHAY and 84 patients had rifampicin resistance and were started on second-line regimens. After excluding them, 5,829 (90%) patients were included in final analysis.

Among patients included in the study, 68% were aged 15–54 years, 77% were males and 2.2% were HIV reactive. 60% of the patients were registered as relapse, 34% as Treatment after default (TAD) and 6% patients as treatment after failure. A total of 5306 (91%) were tested for drug susceptibility with rapid diagnostics before being registered for treatment on category II RNTCP regimen. Of those who were tested, 4933(85%) patients were tested using Line probe assay and remaining using CBNAAT. Of 4933 tested using LPA, 3743 (76%) had valid results for INH resistance of whom 309 (8.3%) were INH resistant. The baseline demographic and clinical characteristics of patients with and without DST at diagnosis are described in Table 1. There were no significant differences between the groups by age, sex, HIV status and type of TB.

**Table 1:** Baseline characteristics of previously-treated smear-positive TB patients, by DST status, registered under RNTCP in Gujarat, India, January-June 2013.

Category	Sub-category	DST done (n = 5,306)		DST not done (n = 523)		*p* value
		N	%	N	%	
**Age groups** *						
	Under 15	33	0.6	6	0.7	0.07
	15–54	4,418	83.3	418	83.0	
	55 or above	846	15.9	99	16.2	
**Sex** *						
	Female	1,211	22.8	125	23.9	
	Male	4,086	77.2	398	76.1	0.59
**HIV status**						
	Non-reactive	4,948	93.3	488	93.3	0.70
	Reactive	117	2.2	9	1.7	
	Unknown	241	4.5	26	5.0	
**Types of registration**						
	Failure	290	5.5	37	7.1	0.09
	TAD	1792	33.8	191	36.5	
	Relapse	3224	60.8	295	56.4	

**(n = 5,829)**

*Age and Sex were not recorded in 9 patients.

TAD-Treatment after default; DST- Drug susceptibility testing; RNTCP-Revised National Tuberculosis Control Programme.

The treatment outcomes of the patients are shown in Table 2. Overall, 71% (4,113) TB patients were reported as successfully treated. Among patients who were tested for DST, 72% were successfully treated as compared to 60% among non-tested. A higher proportion of patients died (13%) and lost to follow up (21%) among non-tested as compared to those who were tested. After excluding patients who were lost to follow up or died during treatment or changed the regimen or transferred out, treatment failure rates were higher among those not tested for drug susceptibility (7.1%) as compared to those who were tested (Table 2). The results of bivariate and multivariate analysis are shown in Table 3. After adjusting for all the other variables (age, sex, HIV status and type of TB), upfront testing for DST was independently associated with treatment outcomes. Patients who did not get tested at diagnosis had a 34% higher risk of unsuccessful outcomes as compared to those who got tested (aRR—1.34; 95% CI 1.20–1.50).

**Table 2:** Treatment outcomes of previously-treated smear-positive TB patients, by DST status, registered under RNTCP in Gujarat, India, January-June 2013.

Treatment outcome	DST Done		DST not done		Total	
	N	%	N	%	N	%
Cured	3716	70.0	302	57.7	4018	68.9
Treatment completed	81	1.5	14	2.7	95	1.6
Died	470	8.9	69	13.2	539	9.2
Default	697	13.1	107	20.5	804	13.8
Failure	259	4.9	24	4.6	283	4.9
Transferred out	49	0.9	3	0.6	52	0.9
Switched to category IV	28	0.5	3	0.6	31	0.5
Outcome not recorded	6	0.1	1	0.2	7	0.1
Total	5,306	100	523	100	5,829	100

**Table 3:** Comparison of treatment outcomes across clinical and demographic factors of previously treated smear-positive TB patients registered in Gujarat from January-June 2013 (n = 5,829).

Category	Sub-Category	Unsuccessful treatment outcome (n = 1,716)		Successful treatment outcome (n = 4,113)		RR (95% CI)	Adjusted RR (95% CI)
		N	%	N	%		
**DST at diagnosis**							
	Yes	1,509	28.4	3,797	71.6	1	1
	No	207	39.6	316	60.4	**1.39 (1.24–1.56)**	**1.34 (1.20–1.50)**
Age groups*							
	Under 15	6	15.4	33	84.6	1	1
	15–54	1,436	29.7	3,400	70.3	1.93 (0.92–4.03)	1.58 (0.76–3.30)
	55 or above	272	28.8	673	71.2	1.87 (0.89–3.93)	1.53 (0.73–3.20)
Sex*							
	Female	305	22.8	1,031	77.2	1	1
	Male	1,409	31.4	3,075	68.6	**1.38 (1.28–1.48)**	**1.39 (1.25–1.55)**
**HIV status**							
	Non-reactive	1,541	28.3	3,895	71.7	1	1
	Reactive	52	41.3	74	58.7	**1.46 (1.18–1.80)**	**1.47 (1.20–1.81)**
	Unknown	123	46.1	144	53.9	**1.63 (1.42–1.86)**	**1.53 (1.34–1.76)**
**Types of registration**							
	Relapse	943	26.8	2,576	73.2	1	1
	TAD	627	31.6	1,356	68.4	**1.18 (1.12–1.25)**	**1.14 (1.05–1.25)**
	Failure	146	44.6	181	55.4	**1.67 (1.46–1.90)**	**1.64 (1.44–1.86)**

*Age and Sex were not recorded in 9 patients.

RR—Relative Risk, CI—Confidence Interval.

RR in bold showed significant difference at p<0.05.

In a subset of patients for whom results of INH susceptibility test were available, treatment outcomes among INH-resistant patients were poorer as compared to those without INH resistance. The difference was statistically significant (p = 0.0003). (Table 4)

**Table 4:** Treatment outcomes of previously-treated smear-positive TB patients, by INH resistance, registered under RNTCP in Gujarat, India, January-June 2013 (N = 3743).

Treatment outcome	INH sensitive		INH resistant	
	N	%	N	%
Cured	2382	69.4	187	60.5
Treatment completed	62	1.8	2	0.6
Died	304	8.9	44	14.2
Default	464	13.5	35	11.3
Failure	190	5.5	2	9.1
Transferred out	18	0.5	6	1.9
Switched to category IV	14	0.4	7	2.3
Total	3434	100	309	100

p = 0.0003 on comparing successful outcomes (cured and treatment completed) and unsuccessful outcomes (death, failure, loss to follow-up or default, transferred out and switched to Category IV regimen).

## Discussion

This is the first study from India showing an association between getting tested for drug susceptibility and treatment outcomes. The study confirms our hypothesis that offering DST at time of diagnosis among PT patients improves their treatment success rates. With increasing test coverage, it has begun to show in the overall programme performance, with an increase in treatment success rates in 2013 to about 71% as compared to previous years which ranged 60%-66% [8–13].

The study reported a high drug susceptibility testing coverage (~90%) among eligible TB patients, one of the better reported coverages from a programmatic setting in India. Previous studies from India and elsewhere in Asia have reported a testing coverage ranging from 39–95% [14–16][17–20]. Possible reasons for such a high coverage may be due to a functional state-wide network of sputum sample collection centres with efficient transportation mechanism, regular listing of all presumptive MDR TB patients and their tracking by RNTCP, knowledgeable and trained programme staff and an excellent system of supply chain management. [21] With increasing expansion and decentralization of DST services expected, the coverage may improve further to reach universal access to testing in the near future.

While treatment success rates were relatively better (72%) among those tested upfront, they are far from what is envisioned (85%) as part of the national strategic plan [22]. With high testing coverage already achieved, it is likely that most of the benefits of this intervention on improving treatment outcomes have been realized. So, any further improvement in treatment outcomes requires additional measures.

One of the observations in the study was that INH resistance was associated with poorer outcomes. Many studies in the past including a meta-analysis have reported similar findings [14,23–26]. Further, the proportion of treatment failure (including those who were switched to MDR-TB treatment) was higher among patients with baseline INH resistance as compared to INH sensitive (11% vs 6%). While we did not have results of DST among the failure cases, many of them are likely to have rifampicin resistance [2] and reflect the extent of acquired rifampicin resistance (ARR). The meta-analysis has also shown that the risk of ARR was higher among those with baseline INH resistance and receiving treatment using intermittent regimens, similar to the one being used by RNTCP [26]. There is growing evidence of poor outcomes even among HIV-negative TB patients, especially among those with baseline INH resistance. This is to be considered seriously given the high prevalence of INH resistance among TB patients in India [27,28].

Further, there have been criticisms about the efficacy of the currently recommended standardized regimen for previously treated patients, given the high prevalence of poly-resistance other than MDR-TB [29]. This calls for a need for DST guided treatment. The national programme has taken steps in this direction (in the form of expert consultations) for developing different regimens for INH resistant TB and the possibility of introducing individualized, DST-guided treatment.

Furthermore, even among patients with INH sensitive TB, loss to follow up is one of the major contributors leading to poorer treatment outcomes. This continues to be one of the core challenges and unmet needs in TB control [30,31] [32]. While direct observation of treatment appears excellent on paper and in trial conditions, there are challenges to its implementation and we need other innovative, patient-support measures beyond DOT including the use of incentives and use of e-health and m-health in improving patient adherence. Unless the barriers of treatment adherence are addressed, the effect of newer strategies will be modest.

We had several strengths. The study had a large sample and was conducted state wide covering all PT patients registered under public health system, thus making the findings generalizable. Standardized definitions were used for clinical variables across all reporting units as personnel responsible for routine reporting had collected data. Standard of care provided was all uniform across all reporting units. We used STROBE guidelines for the reporting of the study. The major limitation of the study was related to reliance on routinely maintained records for all data and the inability to verify the errors that may have happened at the time of recording. Though, we expect such errors to be minimal given the strong structured system of supervision, monitoring and data validation under RNTCP.

To conclude, offering DST at diagnosis improved the treatment outcomes among PT patients. However, even among tested, treatment outcomes remained suboptimal and were related to INH resistance and high loss-to-follow-up. These need to be addressed urgently for further progress.
